# Reporting of surrogate endpoints in randomised controlled trial protocols (SPIRIT-Surrogate): extension checklist with explanation and elaboration

**DOI:** 10.1136/bmj-2023-078525

**Published:** 2024-07-09

**Authors:** Anthony Muchai Manyara, Philippa Davies, Derek Stewart, Christopher J Weir, Amber E Young, Jane Blazeby, Nancy J Butcher, Sylwia Bujkiewicz, An-Wen Chan, Dalia Dawoud, Martin Offringa, Mario Ouwens, Asbjørn Hróbjartsson, Alain Amstutz, Luca Bertolaccini, Vito Domenico Bruno, Declan Devane, Christina D C M Faria, Peter B Gilbert, Ray Harris, Marissa Lassere, Lucio Marinelli, Sarah Markham, John H Powers, Yousef Rezaei, Laura Richert, Falk Schwendicke, Larisa G Tereshchenko, Achilles Thoma, Alparslan Turan, Andrew Worrall, Robin Christensen, Gary S Collins, Joseph S Ross, Rod S Taylor, Oriana Ciani

**Affiliations:** 1 MRC/CSO Social and Public Health Sciences Unit, School of Health and Wellbeing, University of Glasgow, Glasgow, UK; 2 Global Health and Ageing Research Unit, Bristol Medical School, University of Bristol, Bristol, UK; 3 Population Health Sciences, Bristol Medical School, University of Bristol, Bristol, UK; 4 Patient author, UK; 5 Edinburgh Clinical Trials Unit, Usher Institute, University of Edinburgh, Edinburgh, UK; 6 Bristol NIHR Biomedical Research Centre, Bristol, UK; 7 University Hospitals Bristol and Weston NHS Foundation Trust, Bristol, UK; 8 Child Health Evaluative Sciences, Hospital for Sick Children Research Institute, Toronto, ON, Canada; 9 Department of Psychiatry, University of Toronto, Toronto, ON, Canada; 10 Biostatistics Research Group, Department of Population Health Sciences, University of Leicester, Leicester, UK; 11 Women’s College Research Institute, Toronto, ON, Canada; 12 Department of Medicine, University of Toronto, Toronto, ON, Canada; 13 Science, Evidence, and Analytics Directorate, Science Policy and Research Programme, National Institute for Health and Care Excellence, London, UK; 14 Faculty of Pharmacy, Cairo University, Cairo, Egypt; 15 Department of Paediatrics, University of Toronto, Toronto, ON, Canada; 16 AstraZeneca, Mölndal, Sweden; 17 Centre for Evidence-Based Medicine Odense and Cochrane Denmark, Department of Clinical Research, University of Southern Denmark, Odense, Denmark; 18 Open Patient data Explorative Network, Odense University hospital, Odense, Denmark; 19 CLEAR Methods Centre, Division of Clinical Epidemiology, Department of Clinical Research, University Hospital Basel and University of Basel, Basel, Switzerland; 20 Oslo Centre for Biostatistics and Epidemiology, Oslo University Hospital, Oslo, Norway; 21 Bristol Medical School, University of Bristol, Bristol, UK; 22 Department of Thoracic Surgery, IEO, European Institute of Oncology IRCCS, Milan, Italy; 23 IRCCS Galeazzi-Sant’Ambrogio Hospital, Department of Minimally Invasive Cardiac Surgery, Milan, Italy; 24 University of Galway, Galway, Ireland; 25 Health Research Board-Trials Methodology Research Network, University of Galway, Galway, Ireland; 26 Department of Physical Therapy, Universidade Federal de Minas Gerais, Belo Horizonte, Brazil; 27 Fred Hutchinson Cancer Centre, Seattle, WA, USA; 28 St George Hospital and School of Population Health, University of New South Wales, Sydney, NSW, Australia; 29 Department of Neuroscience, Rehabilitation, Ophthalmology, Genetics, Maternal and Child Health, University of Genova, Genoa, Italy; 30 IRCCS Ospedale Policlinico San Martino, Genoa, Italy; 31 Department of Biostatistics and Health Informatics, Institute of Psychiatry, Psychology and Neuroscience, King’s College London, London, UK; 32 George Washington University School of Medicine, Washington, DC, USA; 33 Heart Valve Disease Research Centre, Rajaie Cardiovascular Medical and Research Centre, Iran University of Medical Sciences, Tehran, Iran; 34 Ardabil University of Medical Sciences, Ardabil, Iran; 35 Behyan Clinic, Pardis New Town, Tehran, Iran; 36 University of Bordeaux, Centre d’Investigation Clinique-Epidémiologie Clinique 1401, Research in Clinical Epidemiology and in Public Health and European Clinical Trials Platform & Development/French Clinical Research Infrastructure Network, Institut National de la Santé et de la Recherche Médicale/Institut Bergonié/Centre Hospitalier Universitaire Bordeaux, Bordeaux, France; 37 Charité Universitätsmedizin Berlin, Berlin, Germany; 38 Department of Quantitative Health Sciences, Lerner Research Institute, Cleveland Clinic, Cleveland, OH, USA; 39 McMaster University, Hamilton, ON, Canada; 40 Department of Outcomes Research, Anaesthesiology Institute, Cleveland Clinic, OH, USA; 41 Section for Biostatistics and Evidence-Based Research, the Parker Institute, Bispebjerg and Frederiksberg Hospital, Copenhagen and Research Unit of Rheumatology, Department of Clinical Research, University of Southern Denmark, Odense University Hospital, Odense, Denmark; 42 UK EQUATOR Centre, Centre for Statistics in Medicine, Nuffield Department of Orthopaedics, Rheumatology, and Musculoskeletal Sciences, University of Oxford, Oxford, UK; 43 Department of Health Policy and Management, Yale School of Public Health, New Haven, CT, USA; 44 Section of General Medicine, Department of Internal Medicine, Yale School of Medicine, New Haven, CT, USA; 45 Robertson Centre for Biostatistics, School of Health and Well Being, University of Glasgow, Glasgow, UK; 46 Centre for Research on Health and Social Care Management, Bocconi University, Milan 20136, Italy

## Abstract

Randomised controlled trials often use surrogate endpoints to substitute for a target outcome (an outcome of direct interest and relevance to trial participants, clinicians, and other stakeholders—eg, all cause mortality) to improve efficiency (through shortened duration of follow-up, reduced sample size, and lower research costs), and for ethical or practical reasons. However, their use has a fundamental limitation in terms of uncertainty of the intervention effect on the target outcome and limited information on potential intervention harms. There have been increasing calls for improved reporting of trial protocols that use surrogate endpoints. This report presents the SPIRIT-Surrogate, an extension of the Standard Protocol Items: Recommendations for Interventional Trials (SPIRIT) checklist, a consensus driven reporting guideline designed for trial protocols using surrogate endpoints as the primary outcome(s). The SPIRIT-Surrogate extension includes nine items modified from the SPIRIT 2013 checklist. The guideline provides examples and explanations for each item. We recommend that all stakeholders (including trial investigators and sponsors, research ethics reviewers, funders, journal editors, and peer reviewers) use this extension in reporting trial protocols that use surrogate endpoints. Its use will allow for improved design of such trials, improved transparency, and interpretation of findings when trials are completed, and ultimately reduced research waste.

Randomised controlled trials (referred to as trials in this article) that are well designed, conducted, and reported have a central role in evaluating interventions’ efficacy or effectiveness and potential harms.^1^ The design and conduct of trials should be clearly described in a protocol, including information on ethical considerations, study rationale, methods, and post-trial provisions, among other details.^2^ ^3^ Protocols are essential documents used by various stakeholders: to guide and document study conduct by trial teams; and for appraisal of the planned trial by funders, ethical approval committees, journal editors (and peer reviewers), regulatory and health technology assessment agencies, among other stakeholders.^2^ The inadequate reporting of trials, from the protocol to the final trial results, greatly contributes to the growing issue of research waste.^4^ ^5^ Consequently, reporting guidelines are key interventions to ensure adequate reporting of trial elements, meeting the needs of various stakeholders^2^ and contributing to a reduction in research waste.^5^ The widely used reporting guideline for trial protocols is SPIRIT (Standard Protocol Items: Recommendations for Interventional Trials) 2013, a 33 item checklist.^2^ Although the SPIRIT 2013 checklist has improved the reporting quality of trial protocols,^6^ it might not be sufficient for all types of trials. As a result, SPIRIT extensions including modified or additional items have been developed (eg, SPIRIT-PRO (patient reported outcomes)^7^ and SPIRIT-Outcomes^8^). However, none of the developed extensions provides specific and sufficient guidance for trials that use surrogate endpoints.

Surrogate endpoints are commonly used as substitutes for target outcomes of interest (referred to as target outcomes in this article) in trials, such as all cause mortality, to improve efficiency including shortened duration of follow-up, reduced sample size, and lower research costs, among other reasons.^9^ ^10^ Table 1 provides an example of surrogate endpoints in trials.

**Table 1:** Examples of surrogate endpoints in trials*

No	Item	Example 1^11^	Example 2^12^	Example 3^13^
1	Domain—surrogate endpoint	Blood pressure	Tumour response	Body mass index
2	Measurement variable or specific measurement	Daytime ambulatory systolic blood pressure	Assessed by independent central review according to RECIST (version 1.1) with use of contrast enhanced computed tomography or magnetic resonance imaging	Calculated from weight and height obtained from electronic health record
3	Specific metric	Change from baseline	Value at time point	Change from baseline
4	Method of aggregation	Continuous outcome, mean	Binary outcome, frequency (%)	Per cent from the median body mass index for age and sex
5	Time point	Two months after randomisation	Median 15.3 months	Two years after parent’s consent or enrolment into the study
6	Target outcome(s) as stated by authors	Stroke, coronary heart disease, heart failure, all cause mortality	Overall survival	Diabetes, liver disease, asthma, heart disease, cancer, lower health related quality of life, behaviour problems, psychosocial dysfunction

* First five table rows from core elements of a defined outcome adapted from Butcher et al,^8^ Chan et al,^2^ Mayo-Wilson et al,^14^ and Zarin et al.^15^

Depending on the disease or health area and definitions of a surrogate endpoint, between 17% and 78% of trials use surrogate endpoints as primary outcomes.^16^ ^17^ ^18^ ^19^ However, in the absence of data on target outcomes, their use in trials can be controversial and have important limitations for clinical and policy decision making—that is, failure to provide adequate information on intervention efficacy or effectiveness on the target outcome and harms, mainly owing to small sizes and short follow-up periods associated with surrogate endpoint trials.^20^ Consequently, there have been calls for better reporting of trials that rely on surrogate endpoints, including an explicit statement and rationale for using a surrogate endpoint and consideration of their potential limitations.^19^ ^21^ ^22^ ^23^ Considering the ongoing difficulties in reporting trials that use surrogate endpoints, the SPIRIT/CONSORT-Surrogate project sought to develop extensions for SPIRIT and CONSORT (Consolidated Standards of Reporting Trials) for trials using a surrogate endpoint as a primary outcome (video 1). The CONSORT-Surrogate extension is presented in Manyara et al.^24^ In this article, we report the SPIRIT-Surrogate extension. Table 2 provides a glossary for the terminology used in this extension, including the definition of surrogate endpoints that authors should use in applying this extension.

**Table 2:** Glossary of the terminology used in the SPIRIT-Surrogate extension

Term	Definitions
Composite outcome	Outcome consisting of two or more component outcomes (eg, proportion of participants who died or had a non-fatal stroke). Participants who have experienced any one of the events specified by the components are considered to have experienced the composite outcome.^25^
CONSORT	Consolidated Standards of Reporting Trials. Reporting checklist for completed randomised controlled trials.
CONSORT-Surrogate	Modified CONSORT checklist used to report trials using surrogate endpoints as primary outcomes.
Primary outcome	Predefined outcome that trial teams consider to be the most important and feasible in evaluating the effectiveness, benefits, or harms of an intervention that informs sample size calculation and trial conclusions; sometimes referred to as primary endpoint.^26^ ^27^
Secondary outcome	Outcome(s) usually prespecified in a trial protocol to measure additional intervention effects.^28^
SPIRIT	Standard Protocol Items: Recommendations for Interventional Trials. Checklist used to report trial protocols.
SPIRIT-Surrogate	Modified SPIRIT checklist used to report trial protocols that use surrogate endpoints as primary outcomes.
Surrogate endpoint*†	Endpoint that is used in trials as a substitute for a direct measure of how a patient feels, functions, or survives. A surrogate endpoint does not measure the clinical benefit of primary interest itself, but rather is expected to predict that clinical benefit or harm based on epidemiological, therapeutic, pathophysiological, or other scientific evidence.^9^
Target outcome	Outcome of direct interest and relevance to trial participants, patients, clinicians, trialists, or other stakeholders.^29^

* The SPIRIT/CONSORT-Surrogate extension project research, including an e-Delphi and e-survey, investigated the definition of a surrogate endpoint among different stakeholders: trial participants, clinicians, trialists, regulators, and payers. The results of this research and these definitional considerations are reported in detail elsewhere.^29^

† Other descriptive terms used with “surrogate” are “outcome,” “marker,” “measure,” “observation,” and “parameter”; also referred to as “early,” “replacement,” “proxy,” or “substitute” to describe endpoints, outcomes, measures or markers.

**Summary points**

Randomised controlled trials relying on a surrogate endpoint to replace a target outcome of interest have become increasingly commonplace, particularly in the regulatory approval and health technology assessment of drugs and biologics
Use of surrogate endpoints in trials might be misleading in terms of claims of intervention efficacy or effectiveness on target outcomes, as well as by potentially providing limited information on harms
This article describes the SPIRIT (Standard Protocol Items: Recommendations for Interventional Trials)-Surrogate extension, a guideline to improve reporting of trial protocols using a surrogate endpoint as a primary outcome to consequently inform better patient care, healthcare decisions, and policies
Trial authors, journal editors, and reviewers should use the SPIRIT-Surrogate extension to improve reporting relevant protocols to enhance completeness, transparency, replicability of methods, and usefulness of findings

## Scope and use of SPIRIT-Surrogate

Box 1 provides a summary of the scope and use of the SPIRIT-Surrogate extension. This extension is intended for reporting protocols for all trial types and phases using surrogate endpoints as primary outcomes(s), irrespective of how a surrogate endpoint is defined. Further, its use extends to when surrogate endpoints are part of a composite outcome. Since primary endpoints have a crucial role in evaluating interventions and drawing trial conclusions, the focus of the extension is primarily on this aspect. The extension presents the minimum set of recommended items for reporting, but authors are encouraged to include additional information that enhances the transparency of the planned trial. This extension does not impose any mandate for trial teams to modify their designs or plans to align with the recommended items. Instead, authors should explicitly describe what is planned while strongly considering implementing all items whenever feasible. Box 1 provides more details regarding the scope and application of the extension, and appendix table A1 presents the key methodological considerations in the design and reporting of surrogate endpoints in trial protocols that inform the extension items.

Box 1 **Summary of scope and use of the SPIRIT-Surrogate extension**

**Eligibility for use**

Protocols of trials of all types and phases that use a surrogate endpoint (based on any definition) as the primary outcome(s) in any disease or research area. Includes when a surrogate endpoint is part of a primary composite outcome or composite measure.

**Minimum requirement**

The extension is the minimum set of items to be reported but authors can provide more information for improved transparency and interpretation of findings.

**Surrogate validation methods are out of scope**

The appraisal of surrogate validation methods or metrics to use or cite is out of the scope of this extension. However, researchers are encouraged to read articles on surrogate validation methods in the relevant items.

**Target outcome data**

It is important for trial teams to consider collecting target outcome data (as secondary outcome(s)) even when it is not powered for target outcome(s). Such data are vital in surrogate endpoint validation or capturing potential intervention harms.

**Flexibility in order of reporting items**

Items can be combined or reported in different sections to the items suggested in the extension. The specific item sections are recommendations and not requirements.

**Extrapolation of extension items**

Developed for randomised controlled trials, some items could be relevant to non-randomised trials, observational studies, and other studies using surrogate endpoints.

SPIRIT=Standard Protocol Items: Recommendations for Interventional Trials.

## Development of the SPIRIT-Surrogate extension

Development of the SPIRIT-Surrogate extension, which was carried out concurrently with CONSORT-Surrogate extension, followed four sequential phases drawing on the EQUATOR (Enhancing the Quality and Transparency Of health Research) Network guidance for developing health reporting guidelines.^30^ The development was registered on the EQUATOR Network website,^31^ and the protocol was published.^32^ Phase 1 involved literature reviews aimed at synthesising reporting items of trials using surrogate endpoints from current literature and identifying surrogate content experts (scoping review); and identifying investigators of recent trials using surrogate endpoints as primary outcomes for the invitation to an e-Delphi survey (targeted review). The protocol for the literature reviews has been published elsewhere.^33^ The scoping review search was conducted between March and May 2022, and 90 documents were included after screening. Data on definitions, limitations, acceptability, and guidance were extracted and used to generate 17 trial reporting items; the findings of the scoping review including the 17 generated items have been published elsewhere.^20^ After a project team discussion, nine items were taken forward for rating in the e-Delphi survey.

Phase 2 of the study involved a two round, e-Delphi survey to assess the importance of potential reporting items. The survey used a 9 point Likert scale (1-3: not important, 4-6: important but not critical, 7-9: critical). It was conducted using the DelphiManager software (version 5.0) maintained by the COMET (Core Outcome Measures in Effectiveness Trials) initiative, https://www.cometinitiative.org/delphimanager/. The first round was open from 24 August to 10 October 2022, while the second round took place from 31 October to 11 December 2022. Participants were identified through various strategies: contacting authors of relevant articles from the literature reviews; project team professional contacts; and calls for participants made in conferences and meetings, social media, and distributed through professional organisations and networks (supplementary material (acknowledgments)).

A total of 212 eligible participants registered to participate in the survey, with 195 (92%) providing ratings in the first round and 176 (83%) in the second round. The participants were drawn from 30 countries and represented a diverse group of stakeholders, including trial investigators, methodologists and managers, clinicians and allied health professionals, statisticians, surrogate content experts, journal editors, patient and public partners, regulators, experts on health technology assessments, ethics committees, and funding panel members. Participation was multidisciplinary, with representation from over 26 disease and research areas (appendix tables A2, A3, A4, and A5 provides characteristics of participants).

Consensus thresholds were predefined on the basis of previous extensions, and were categorised as follows: consensus for inclusion (≥70% score of 7-9 and <15% score of 1-3), consensus for exclusion (≥70% score of 1-3 and <15% score of 7-9), and no consensus for inclusion or exclusion (failure to meet either threshold).^32^ In the first round, nine items were rated for the SPIRIT-Surrogate extension and 10 items in the second round (an additional item was suggested by a participant in the first round). Six items reached the consensus thresholds in the first round and one more item in the second round. However, no consensus was reached for three items after both rounds. Appendix table A6 provides further details.

Phase 3 of the study involved a hybrid consensus meeting held on 13-14 March 2023, both at the University of Glasgow, Glasgow, UK and via Zoom. The meeting delegates consisted of the project team members (n=13) and a selected group of stakeholders who had participated in the e-Delphi survey (n=20). During the meeting, the three items that did not reach consensus in the e-Delphi survey were discussed and subjected to voting using the www.mentimeter.com platform. Consensus was predefined as at least 70% of the participants voted to either include or exclude an item. As a result, all three items achieved consensus for inclusion (appendix table A7). For the items that reached consensus, the meeting delegates further discussed merging and refining the wording of final checklist items and considered free-text comments received from the e-Delphi surveys.

Phase 4 has been ongoing since the start of the project and involves knowledge translation, which encompasses the dissemination and implementation of the extensions. Dissemination efforts have included publishing short articles to promote the project,^34^ ^35^ ^36^ ^37^ ^38^ publication of protocols,^32^ ^33^ and delivering presentations at various meetings and conferences. Finally, the completed checklist was pilot tested by eight trial investigators with experience in conducting at least one trial by providing them with published protocols and asking them to note whether extension items were reported. There was clarity in all items, and no changes were made following the pilot exercise.

## Structure of SPIRIT-Surrogate extension

The extension comprises of the checklist, explanation, and elaboration sections to clarify on modified items, and examples of their use in published protocols. When items remain unmodified, readers should use the SPIRIT 2013 checklist.^2^ We draw from 11 published protocols to provide at least one example of reporting for each of the nine SPIRIT-Surrogate extension items. These protocols were identified from a targeted review of trial protocols published between January 2017 and June 2022 in *BMJ Open* and *Trials* journals. Examples are quoted verbatim and cited, while references within the examples are denoted using the term “ref” in superscript. Some examples are supplemented by added terms in square brackets and recommendations at the end of the quotes to improve their use as exemplars. Abbreviations have also been spelt out in the examples where necessary. Using examples from the protocols does not constitute our support of the trial or endorsement of interventions evaluated.

Furthermore, it is impossible to identify and exhaustively provide examples from all disease and research areas that should use this extension. Trial teams should therefore use the examples to guide how the items can be implemented in their research areas. Nevertheless, the identifying examples on nearly all extension items demonstrates the feasibility of implementing the extensions in relevant protocols.

Despite extensive efforts, including a review of protocols from targeted review and solicitation of exemplars from colleagues, we could not find an exemplar implementing one item: informing participants that the trial will use a surrogate endpoint. Therefore, we modify a published protocol to show how this item can be reported (item 26a.1). Given the nature of this item, patient and public partners who are coauthors of this extension (DS, SM, RH, AW) conceptualised and helped with drafting a potential structure for implementing the item in participant information sheets along with examples. 

## SPIRIT-Surrogate extension

Table 3 compares the SPIRIT 2013 checklist and the extension items in the SPIRIT-Surrogate checklist. Appendix 2 presents a combined SPIRIT 2013 and SPIRIT-Surrogate checklist; this table is downloadable as a fillable document.

**Table 3:** Comparison of the SPIRIT 2013 items and SPIRIT-Surrogate extension items

Section/item	Item No	SPIRIT 2013 items	SPIRIT-Surrogate items
**Administrative information**			
Title	1	Descriptive title identifying the study design, population, interventions, and, if applicable, trial acronym	—
Trial registration	2a	Trial identifier and registry name. If not yet registered, name of intended registry	—
	2b	All items from the World Health Organization Trial Registration Data Set	—
Protocol version	3	Date and version identifier	—
Funding	4	Sources and types of financial, material, and other support	—
Roles and responsibilities	5a	Names, affiliations, and roles of protocol contributors	—
	5b	Name and contact information for the trial sponsor	—
	5c	Role of study sponsor and funders, if any, in study design; collection, management, analysis, and interpretation of data; writing of the report; and the decision to submit the report for publication, including whether they will have ultimate authority over any of these activities	—
	5d	Composition, roles, and responsibilities of the coordinating centre, steering committee, endpoint adjudication committee, data management team, and other individuals or groups overseeing the trial, if applicable (see item 21a for data monitoring committee)	—
**Introduction **			
Background and rationale	6a	Description of research question and justification for undertaking the trial, including summary of relevant studies (published and unpublished) examining benefits and harms for each intervention	8.1 State (a) that the primary outcome is a surrogate endpoint, and (b) the target outcome(s) whose intervention effect is being substituted for
	6b	Explanation for choice of comparators	
Objectives	7	Specific objectives or hypotheses	
Trial design	8	Description of trial design including type of trial (eg, parallel group, crossover, factorial, single group), allocation ratio, and framework (eg, superiority, equivalence, non-inferiority, exploratory)	
**Methods: participants, interventions, and outcome **			
Study setting	9	Description of study settings (eg, community clinic, academic hospital) and list of countries where data will be collected. Reference to where list of study sites can be obtained	—
Eligibility criteria	10	Inclusion and exclusion criteria for participants. If applicable, eligibility criteria for study centres and individuals who will perform the interventions (eg, surgeons, psychotherapists)	—
Interventions	11a	Interventions for each group with sufficient detail to allow replication, including how and when they will be administered	—
	11b	Criteria for discontinuing or modifying allocated interventions for a given trial participant (eg, drug dose change in response to harms, participant request, or improving/worsening disease)	—
	11c	Strategies to improve adherence to intervention protocols, and any procedures for monitoring adherence (eg, drug tablet return, laboratory tests)	—
	11d	Relevant concomitant care and interventions that are permitted or prohibited during the trial	—
Outcomes	12	Primary, secondary, and other outcomes, including the specific measurement variable (eg, systolic blood pressure), analysis metric (eg, change from baseline, final value, time to event), method of aggregation (eg, median, proportion), and time point for each outcome. Explanation of the clinical relevance of chosen efficacy and harm outcomes is strongly recommended	12.1 State the practical or scientific reason(s) for using a surrogate endpoint as a primary outcome
			12.2 State what other surrogate endpoints were considered and why the current one(s) were chosen
			12.3 Justification for selected surrogate endpoint: (a) evidence (or lack of evidence) of surrogate endpoint validation; and (b) evidence (or lack of evidence) of validity being specific to the context used (eg, intervention, disease, population)
Participant timeline	13	Time schedule of enrolment, interventions (including any run-ins and washouts), assessments, and visits for participants. A schematic diagram is highly recommended	—
Sample size	14	Estimated number of participants needed to achieve study objectives and how it was determined, including clinical and statistical assumptions supporting any sample size calculations	14.1 Clarify if the sample size will be estimated to demonstrate that a minimum effect on the surrogate endpoint would be predictive of a benefit on the target outcome(s)
Recruitment	15	Strategies for achieving adequate participant enrolment to reach target sample size	
**Methods: assignment of interventions (for controlled trials) **			
Allocation:			
Sequence generation	16a	Method of generating the allocation sequence (eg, computer-generated random numbers), and list of any factors for stratification. To reduce predictability of a random sequence, details of any planned restriction (eg, blocking) should be provided in a separate document that is unavailable to those who enrol participants or assign interventions	—
Allocation concealment mechanism	16b	Mechanism of implementing the allocation sequence (eg, central telephone; sequentially numbered, opaque, sealed envelopes), describing any steps to conceal the sequence until interventions are assigned	—
Implementation	16c	Who will generate the allocation sequence, who will enrol participants, and who will assign participants to interventions	—
Blinding (masking)	17a	Who will be blinded after assignment to interventions (eg, trial participants, care providers, outcome assessors, data analysts), and how	—
	17b	If blinded, circumstances under which unblinding is permissible, and procedure for revealing a participant’s allocated intervention during the trial	—
**Methods: data collection, management, and analysis **			
Data collection methods	18a	Plans for assessment and collection of outcome, baseline, and other trial data, including any related processes to promote data quality (eg, duplicate measurements, training of assessors) and a description of study instruments (eg, questionnaires, laboratory tests) along with their reliability and validity, if known. Reference to where data collection forms can be found, if not in the protocol	—
	18b	Plans to promote participant retention and complete follow-up, including list of any outcome data to be collected for participants who discontinue or deviate from intervention protocols	—
Data management	19	Plans for data entry, coding, security, and storage, including any related processes to promote data quality (eg, double data entry; range checks for data values). Reference to where details of data management procedures can be found, if not in the protocol	—
Statistical methods	20a	Statistical methods for analysing primary and secondary outcomes. Reference to where other details of the statistical analysis plan can be found, if not in the protocol	—
	20b	Methods for any additional analyses (eg, subgroup and adjusted analyses)	20b.1 State what the plans are to conduct subsequent analyses/studies to verify current findings on the target outcome(s)
	20c	Definition of analysis population relating to protocol non-adherence (eg, as randomised analysis), and any statistical methods to handle missing data (e.g., multiple imputation)	—
**Methods: monitoring**			
Data monitoring	21a	Composition of data monitoring committee (DMC); summary of its role and reporting structure; statement of whether it is independent from the sponsor and competing interests; and reference to where further details about its charter can be found, if not in the protocol. Alternatively, an explanation of why a DMC is not needed	—
	21b	Description of any interim analyses and stopping guidelines, including who will have access to these interim results and make the final decision to terminate the trial	—
Harms	22	Plans for collecting, assessing, reporting, and managing solicited and spontaneously reported adverse events and other unintended effects of trial interventions or trial conduct	22.1 Comment on whether the trial design (including sample size and follow-up period), given the use of a surrogate endpoint, adequately captures the potential harms of the intervention being tested
Auditing	23	Frequency and procedures for auditing trial conduct, if any, and whether the process will be independent from investigators and the sponsor	—
**Ethics and dissemination**			
Research ethics approval	24	Plans for seeking research ethics committee/institutional review board (REC/IRB) approval	—
Protocol amendments	25	Plans for communicating important protocol modifications (eg, changes to eligibility criteria, outcomes, analyses) to relevant parties (eg, investigators, REC/IRBs, trial participants, trial registries, journals, regulators)	—
Consent or assent	26a	Who will obtain informed consent or assent from potential trial participants or authorised surrogates, and how (see item 32)	26a.1 State whether and how trial participants will be engaged and informed before enrolment that the trial was designed to evaluate an intervention’s effect using a surrogate endpoint
	26b	Additional consent provisions for collection and use of participant data and biological specimens in ancillary studies, if applicable	—
Confidentiality	27	How personal information about potential and enrolled participants will be collected, shared, and maintained in order to protect confidentiality before, during, and after the trial	—
Declaration of interests	28	Financial and other competing interests for principal investigators for the overall trial and each study site	—
Access to data	29	Statement of who will have access to the final trial dataset, and disclosure of contractual agreements that limit such access for investigators	—
Ancillary and post-trial care	30	Provisions, if any, for ancillary and post-trial care, and for compensation to those who suffer harm from trial participation	—
Dissemination policy	31a	Plans for investigators and sponsor to communicate trial results to participants, healthcare professionals, the public, and other relevant groups (eg, via publication, reporting in results databases, or other data sharing arrangements), including any publication restrictions	—
	31b	Authorship eligibility guidelines and any intended use of professional writers	—
	31c	Plans, if any, for granting public access to the full protocol, participant level dataset, and statistical code	31c.1 If surrogate and target outcome data will be collected in the trial, state the open access arrangements for the data for future secondary research
**Appendices **			
Informed consent materials	32	Model consent form and other related documentation given to participants and authorised surrogates	—
Biological specimens	33	Plans for collection, laboratory evaluation, and storage of biological specimens for genetic or molecular analysis in the current trial and future use in ancillary studies, if applicable	—

Appendix 2 presents a combined SPIRIT 2013 and SPIRIT-Surrogate checklist, which can be downloaded and completed separately.

IRB=institutional review board; REC=research ethics committee; SPIRIT=Standard Protocol Items: Recommendations for Interventional Trials.

## Section 1: Administrative information (unmodified)

All items in this section (items 1, 2a, 2b, 3, 4, 5a, 5b, 5c, and 5d) are unmodified—see SPIRIT 2013.^2^

## Section 2: Introduction (extended)

See SPIRIT 2013^2^ for item 6a and 6b (background and rationale), and 7 (objectives).

### Trial design

#### SPIRIT 2013 item 8

Description of trial design including type of trial (eg, parallel group, crossover, factorial, single group), allocation ratio, and framework (eg, superiority, equivalence, non-inferiority, exploratory).

See SPIRIT 2013.^2^

#### SPIRIT-Surrogate extension item 8.1

State (a) that the primary outcome is a surrogate endpoint, and (b) the target outcome(s) whose intervention effect is being substituted for. 

This item can be reported under items 6a, 7, or 8.

### Examples of SPIRIT-Surrogate extension item 8.1

#### Example 1

“In different models of hypertension only intermittent hypoxia, which is the main stimuli in OSA [muscle sympathetic nerve activity], causes neurogenesis modulation in hippocampus ^ref^. In humans, intermittent hypoxic exposure induces after 2 and 4 weeks an increase in daytime MSNA [muscle sympathetic nerve activity] ^refs^. This increase in sympathetic tone was suggested in the early ‘90s as a mechanism of hypertension in OSA ^refs^. Therefore, MSNA measurement is of particular interest in showing the effect of OSA treatment as a surrogate marker of cardiovascular outcomes”^39^. (Authors should be specific on the target outcome(s) being substituted for.)

#### Example 2

**“**Primary objective: As a surrogate parameter for clinical improvement and primary outcome we will use the PaO2/FiO2 [arterial oxygen partial pressure/fractional inspired oxygen] (P/F) ratio.”^40^

#### Example 3

“Trial design: The mean SOFA [sequential organ failure assessment] score (at least two individual values) during treatment and subsequent intensive care of up to 14 days is used as surrogate outcome (primary endpoint). Secondary outcome measures include 30- and 90-day mortality.”^41^ (Authors should be specific on the target outcome(s) being substituted for.)

#### Explanation

The introduction of a trial protocol summarises current evidence and knowledge gaps being filled.^42^ ^43^ It allows journal editors and reviewers to assess the importance of the planned trial.^42^ Background sections in protocols mirror subsequent introduction sections of trial reports to a large extent but could be more lengthy to explain the rationale of a trial.^42^ Therefore, authors must be explicit about using a surrogate endpoint and the target outcome being predicted or substituted for. Authors can decide to report the item under relevant items such as with item 6a of the SPIRIT 2013 (example 1), item 7 (example 2), or item 8 (example 3). Wherever it is reported, authors should ensure they are explicit and detailed on both the surrogate (part 8.1(a) of the item) and target outcomes (part 8.1(b) of the item).

## Section 3a: Methods—participants, interventions, and outcomes

### Outcomes (extended)

#### SPIRIT 2013 item 12

Primary, secondary, and other outcomes, including the specific measurement variable (eg, systolic blood pressure), analysis metric (eg, change from baseline, final value, time to event), method of aggregation (eg, median, proportion), and time point for each outcome. 

Explanation of the clinical relevance of chosen efficacy and harm outcome is strongly recommended (see SPIRIT 2013^2^ and SPIRIT-Outcomes extension^8^).

#### SPIRIT-Surrogate extension item 12.1

State the practical or scientific reason(s) for using a surrogate endpoint as a primary outcome.

#### SPIRIT-Surrogate extension item 12.2

State what other surrogate endpoints were considered and why the current one(s) were chosen.

#### SPIRIT-Surrogate extension item 12.3

Justification for selected surrogate endpoint: (a) evidence (or lack thereof) of surrogate endpoint validation; and (b) evidence (or lack thereof) of validity being specific to the context used (eg, intervention, disease, population).

### Examples of SPIRIT-Surrogate item 12.1

#### Example 1: study mechanisms of action

“Our study is focused on cardiovascular surrogate parameters, such as CFR [coronary flow reserve] function, which cannot replace outcome trials but can provide insights into the potential mechanisms of the cardiovascular effects of CXCR2 [chemokine receptor] inhibition.”^44^

#### Example 2: inform conduct of phase 3 trial

“The short-term endpoint [surrogate endpoint] of MRD [minimal residual disease] negativity will be assessed to determine whether continuation to the phase III part of the trial is worthwhile.”^45^ (We have added the term “surrogate endpoint” and recommend its use.)

#### Explanation

Considering limitations associated with surrogate endpoints,^20^ authors should explain to readers the practical or scientific rationale for using them. A primary reason for the use of surrogate endpoints is trial efficiency: smaller sample sizes and shorter follow-up periods than those when using target outcomes. This efficiency can be ideal for early phase trials that aim to inform future trials powered on target outcomes. Further, trials of primary disease prevention can require a long follow-up period to observe or measure the target outcome, while trials of rare diseases often have access to small trial populations.^20^ In regulatory approval settings, surrogate endpoints have been widely used as part of expedited or accelerated approval of interventions for conditions with high unmet medical need for serious or life threatening diseases.^9^ ^46^ Also, in certain interventional contexts, target outcomes might not be ideal—for example, participant reported outcomes in paediatric trials might not be possible^47^ and observer reported outcomes are needed in newborn babies or very young children (age <7 years). The practical or scientific reasons for using surrogate endpoints highlighted here and elsewhere^20^ might not be exhaustive.

Reporting this item gives readers a preliminary justification for using surrogate endpoint(s) as a primary outcome and contextualises the trial’s significance. Nevertheless, authors should still discuss the justification of the chosen surrogate endpoint (see next items).

### Example of SPIRIT-Surrogate item 12.2

“The study relies on BMI [body mass index] as the only [surrogate endpoint] measure of intervention effectiveness. We considered using other clinical biomarkers (e.g., Hemoglobin A1c (HbA1c) or blood pressure) in addition to BMI, but they are frequently missing in EHR [electronic health record] data, and the study or the families themselves may have had to cover their cost. We also considered using self-reported dietary intake measures or accelerometers to measure physical activity. However, given the budget, these were not financially feasible. We also had concerns that adding assessments would impose a burden that might discourage enrolment into the trial and subsequent retention.”^13^ (We have added the words in square brackets in the example and recommend their use when reporting the item.)

#### Explanation

Selection of outcomes is a key step in trial design.^48^ ^49^ The SPIRIT 2013 checklist recommends a complete definition of outcomes, an explanation of the rationale for selected outcomes, and keeping the number of primary outcomes to a minimum.^2^ Over the past decade, development of the core outcome sets has provided a collection of consensus driven outcomes that can be measured across intervention effectiveness trials.^50^ ^51^ They have been a useful contribution, but core outcome sets have yet to be developed for all disease areas, and types of trial design (eg, early phase core outcome sets are lacking) and sometimes primary outcomes might not be within the core outcome set.^51^ For example, early phase trials might use surrogate endpoints for efficacy rather than outcome(s) from core outcome sets.^52^

When using outcomes that are not part of a consensus driven collection, such as the strict procedures used to identify outcomes in a core outcome set, there is a risk of selecting a surrogate endpoint without a justification (ie, cherry picking). Trial teams should therefore be explicit on alternative surrogate endpoints considered and on what factors informed the choice of current endpoints. This conduct would improve transparency and provide insights into the strengths and weaknesses of the surrogate endpoint(s) used for future interpretation of findings. The reporting of this item will vary depending on the surrogate endpoint(s) used. For some, the selection will be based on practical reasons, such as in the example provided, and could therefore be reported in combination with the previous item (item 12.1). In other cases, the choice of a surrogate endpoint(s) could be based on scientific rationale, such as effect sizes or surrogate endpoint validity. It can, therefore, be reported in combination with the next item (item 12.3).

### Examples of SPIRIT-Surrogate item 12.3

#### Example 1

“The primary outcome of the IMPROVE-CKD study is change in large arterial compliance (as measured by carotid-femoral PWV [pulse wave velocity]) at 96 weeks after randomisation to lanthanum carbonate or placebo. Hyperphosphatemia has been associated with reduced arterial compliance, and multiple studies have reported a positive relationship between serum phosphate and PWV ^refs^. PWV has been used to measure arterial compliance and is considered to be a valid surrogate for cardiovascular morbidity and mortality ^refs^. PWV also correlates with CKD [chronic kidney disease] stage and increases as CKD progresses ^refs^.”^53^ (Use of evidence from observational studies is not sufficient to justify validity of a surrogate endpoint, see the explanation of item 12.3 for more details.)

#### Example 2

“The primary efficacy endpoint is the change in daytime ambulatory systolic blood pressure from baseline to 2 months. Systolic blood pressure is a validated surrogate endpoint for prediction of cardiovascular events and mortality based on a meta-analysis of 123 blood pressure lowering drug trials, with 613,815 participants demonstrating a strong association between the treatment effect of systolic blood pressure and cardiovascular events ^ref^. Specifically meta-regression showed relative risk reductions for major cardiovascular disease events (P<0.0001), stroke (P<0.0001), heart failure (P<0.0001), and all-cause mortality (P=0.014) to be proportional to the magnitude of the systolic blood pressure reduction achieved. However, risk reductions for various diseases differed across drug classes more evidence is needed to establish that validity of blood pressure lowering to predict for benefit in cardiovascular events and mortality holds when renal denervation is used.” (This example was written by the extension authors from a published trial^11^ and using the meta-analysis^54^ cited by the trial to show reporting of trial level validity; see the explanation of item 12.3 for more details.)

#### Explanation

Surrogate endpoints should be validated before their use. Validation is the process of ascertaining that the intervention’s effect on the surrogate endpoint predicts the intervention’s effect on the target outcome.^55^ ^56^ A detailed discussion of surrogate validation is beyond the scope of this extension: nevertheless, we signpost readers to articles on surrogate endpoint validation methods,^55^ ^56^ ^57^ ^58^ ^59^ ^60^ ^61^ ^62^ ^63^ ^64^ ^65^ frameworks for evaluating evidence of validity,^21^ ^66^ ^67^ ^68^ and a recent checklist to report the surrogate endpoint validation process.^69^

Briefly, surrogate validation should show both a strong association of the surrogate endpoint and target outcome (the so-called individual level association), and show that the treatment effect on the surrogate is strongly correlated with the treatment effect on the target outcome (the so-called trial level association).^55^ ^56^ Example 1 falls short of this optimal level of evidence, because it cites correlation evidence between pulse wave velocity (surrogate endpoint) and cardiovascular morbidity and mortality (target outcomes). In contrast, example 2 uses a meta-analysis of randomised controlled trials to justify a treatment effect association between the surrogate endpoint (systolic blood pressure) and target outcomes (cardiovascular events and all cause mortality). To fully judge the strength for validity of a surrogate endpoint, authors should provide some key meta-regression metrics: the slope coefficient (and 95% confidence intervals) of the linear association between the treatment effect of the surrogate and the target outcome, the strength of the association such as Spearman’s correlation coefficient (ρ) or R^2^ at the individual level and trial level, and the surrogate treatment effect or prediction intervals (see item 14.1 below). Illustration of these metrics for blood pressure and the risk of cardiovascular events can be found in the article by Lassere et al.^70^

Surrogate endpoint validation in trials is inadequately reported. An audit of 626 trials published in 2005 and 2006 found that 37 (34%) of the 109 trial reports that used a surrogate endpoint as a primary outcome discussed the surrogate validity.^71^ In cancer, where several surrogate endpoint validation studies have been published, a systematic review indicated relatively low levels of validity for treatment effects at the trial level between surrogate endpoints and target outcomes. About half (52%) of surrogate endpoints used in trials demonstrated a low study level correlation (r ≤0.7) with the treatment effect on the surrogate endpoint, and only 23% were highly correlated (r ≥0.85) with the treatment effect on the target outcome of overall survival.^72^ Surrogate validation models often allow for prediction of the treatment effect on the target outcome in new trials for which the effect on the surrogate endpoint has been estimated. It is therefore important to quantify the accuracy of the predictions made.^69^ Leave-one-out cross validation and external validation, with new trials published after the model was fitted or trials whose individual patient data were not available for model estimation, are essential to assess the model’s predictive performance and calibration.^73^ This observation highlights the need for being explicit about surrogate validity evidence or about the lack of it when surrogate endpoints are used. Over time, many surrogate endpoint statistical approaches for validation have been proposed (box 2).^62^ ^78^ The validation approach underpinning the selection of the surrogate endpoint should be clearly presented, including, when possible, the prediction equation being considered to later allow for the prediction of the effect on the target outcome.

Box 2 **Summary of statistical approaches for surrogate endpoint validation**

Selected and non-exhaustive statistical methods and general approaches for evaluating the validity of surrogate endpoints in the assessment of treatment efficacy that have emerged over the last four decades.

**Prentice’s criteria^61^**

In pioneering work published in 1989, Prentice proposed three criteria for valid hypothesis testing extrapolation (rejecting the null hypothesis of no treatment effect on the surrogate endpoint implies rejecting the null hypothesis of no treatment effect on the target outcome):

- The effect of the surrogate endpoint on the true endpoint does not vary with randomisation group;
- The surrogate endpoint affects the true endpoint;
- The effect of treatment on the surrogate endpoint changes the average effect of treatment on true endpoint.

The Prentice criteria remains conceptually important but of limited usefulness in practice.

**Principal stratification^74^**

This method maintains that causal effects should be the basis for surrogate endpoint evaluations, where the causal effect is a comparison between treatment groups of the potential outcomes on the same set of individuals. Two requirements are needed for surrogate validity: causal necessity, which requires that an effect of treatment on the target outcome can only exist if treatment has also affected the surrogate; and statistical generalisability, which requires good predictive performance of the surrogate for the target outcome in a future study in which only the surrogate is observed.

**Meta-analytical regression based approach^55^ ^75^**

This approach relies on two stage, joint modelling of the surrogate and target outcome in a multi-trial (randomised trials) setting. Surrogacy is established on the basis of the coefficient of determination between the surrogate and target outcome at the individual patient level (individual level R^2^), and the coefficient of determination between the treatment effect on the surrogate and on the target outcome at the trial level (trial level R^2^). Alternatively, the surrogate threshold effect has been proposed as a practical measure to define the minimum level of treatment effect required on the surrogate to conclude that a significant treatment effect would also be present on the target outcome.^76^ Extensions of these meta-analytical methods based on information theory have been proposed as the preferred approach under the causal association paradigm.^77^

**Bayesian approaches**

While a bayesian approach will be readily applicable to all the methodologies outlined above, the most commonly used models are the meta-analytical fixed (independent) effects model proposed by Daniels and Hughes^78^ and a bayesian random effects meta-analysis to model trial level effects on the target outcome and surrogate endpoint.^59^ More recently, bayesian multivariate meta-analytical methods to take into account the association between the treatment effects on the surrogate and target outcomes have been proposed specifically for regulatory and reimbursement decision making.^59^

Validity for trial level treatment effects established in a particular trial context (eg, sufficiently similar population, intervention, disease, control, and setting) might not be extrapolated to another.^20^ For instance, a systematic review of studies evaluating the validity of progression-free survival as a surrogate endpoint for overall survival reported that trial level validity differed across intervention evaluated, cancer localisation, and cancer stage.^79^ Body mass index reduction that predicts health and mortality benefit depends on the disease or obesity related complication, the person’s age, and their baseline obesity level.^80^ ^81^ Therefore, trial protocols should support the validity of any surrogate endpoints used in relevant contexts, such as in example 2, where surrogate endpoint validity for different diseases is mentioned but lack of evidence on being specific to intervention being tested is highlighted.

### Sample size (extended)

#### SPIRIT 2013 item 14

Estimated number of participants needed to achieve study objectives and how it was determined, including clinical and statistical assumptions supporting any sample size calculations.

See SPIRIT 2013.^2^

#### SPIRIT-Surrogate extension item 14.1

Clarify if the sample size will be estimated to demonstrate that a minimum effect on the surrogate endpoint would be predictive of a benefit on the target outcome(s).

### Example of SPIRIT-Surrogate item 14.1

#### Example 1

“Calculations of the required sample size were conducted for our primary analysis. Based on our pilot study results, we expect a between-group difference of 25 min per week (SD [standard deviation]=50) at 3 months, yielding an ES [effect size] of 0.5. A similar ES is expected at 5.5 months assuming weather will not limit walking outdoors [the surrogate endpoint]. Based on accelerometery/GPS [global positioning system] data that we collected 6 months postbaseline during cooler weather conditions, a decrease in ES of ~10% to 0.4 (between group difference 20 min per week, SD=50) is expected for the 0-12-month comparison. A 20 min group difference exceeds 10% of the weekly physical activity recommendation and would help move seniors from a sedentary to a low active classification associated with higher HRQL [health related quality of life; the target outcome] ^ref^. Thus, sample size estimation will be based on detecting the smaller ES of 0.4. In the pilot study, there was no attrition from 0 months to 6 months; however, we have allowed for a 5% attrition rate from 0 months to 6 months in the proposed study, and a 20% attrition rate from 0 months to 12 months based on rates observed in studies of group-based physical activity interventions ^ref^. Given an ES of 0.4, type I error level=0.05, type II error level=0.20, equal number of participants/group and a 20% attrition rate, a total sample size of 240 is required.”^82^ (We have added the words in square brackets in the example and we recommend their use when reporting the item.)

#### Example 2 

“The assumptions for the power calculation (threshold of a 40-m increase as the [surrogate threshold effect] minimal clinically important improvement in 6-minute walk test distance, with an SD [standard deviation] of 80m) [will be based] based on (1) a meta-regression of prior randomized clinical trials in patients with pulmonary arterial hypertension ^ref^ (due to the lack of such data in patients with HFpEF [heart failure with preserved ejection fraction]) and (2) clinical consensus among members of the trial’s steering committee.”^83^ (We have added the words in square brackets to this example, which is from a completed trial that has been used to show its use in a protocol. We recommend using the term “surrogate threshold effect” rather than “minimal clinically important improvement,” which is consistent with the cited surrogate validation study.)

#### Explanation

Trial sample size determination should be appropriately justified and adequately reported including information on the target effect size and allowance for trial sample attrition.^2^ ^43^ ^84^ Trials using a surrogate endpoint as the primary outcome should consider their choice of a target effect size based on surrogate validity metrics. A common minimum effect on the surrogate endpoint predicting a benefit on the target outcome derived using trial data are known as a surrogate threshold effect. Nevertheless, surrogate threshold effects are not available for all surrogate endpoints and other metrics of surrogate validity can be used. For instance, in the example provided,^82^ the authors cite a dose-response analysis between walking and physical function from a cross sectional study.^85^

In some cases, authors might not be able to use any surrogate endpoint validity metrics to calculate the sample size because of no prior research on surrogate validation. Therefore, authors should clarify when surrogate validity metrics were not used. Furthermore, given that surrogate endpoints are mainly used improve trial efficiency (ie, with smaller samples compared with trials using target outcomes), authors are encouraged to determine the sample size for both the surrogate endpoint and target outcome. If the sample size based on treatment effect on the target outcome is similar (or lower) compared to that of using surrogate endpoint, then the choice of surrogate endpoint as the primary outcome should be sufficiently justified. Additionally, if the target outcome data were collected, authors might consider validating the surrogate endpoint prospectively or ensuring that others could access the data for similar research (see item 31c.1 below).

Finally, at the trial reporting stage, whether validity metrics are used or not, authors will need to interpret findings in the context of using a surrogate endpoint and its known validity, including how the predicted effect on the target outcome and the uncertainty in this, reflected by its confidence interval, will be derived (see CONSORT-Surrogate).

## Section 3c: Methods—data collection, management, and analysis

### Statistical methods (extended)

#### SPIRIT 2013 item 20a

Statistical methods for analysing primary and secondary outcomes. Reference to where other details of the statistical analysis plan can be found, if not in the protocol.

See SPIRIT 2013.^2^

#### SPIRIT 2013 item 20b

Methods for any additional analyses (eg, subgroup and adjusted analyses).

See SPIRIT 2013.^2^

#### SPIRIT-Surrogate extension item 20b.1

State what the plans are to conduct subsequent analyses/studies to verify current findings on the target outcome(s).

#### SPIRIT 2013 item 20c

Definition of analysis population relating to protocol non-adherence (eg, as randomised analysis), and any statistical methods to handle missing data (eg, multiple imputation).

See SPIRIT 2013.^2^

### Example of SPIRIT-Surrogate item 20b.1

#### Example 1: Reporting ongoing trial

“Given the paucity of novel therapies and the many clinical trials that have failed to show efficacy for SLE [systemic lupus erythematosus], a combination of biological therapies with complementary effects given in succession may be required to control the disease. If the results of this trial are promising, a larger trial will be required of sufficient power to detect improved clinical outcomes. Indeed, a larger trial is already underway (BLISS BELIEVE NCT03312907) testing whether rituximab given after belimumab confers an additional benefit compared with belimumab alone ^ref^.”^86^

#### Example 2: Analysis of target outcome which is collected as a secondary outcome

“All secondary endpoint analyses comparing randomised participants will be assessed at the same time as the primary [surrogate] endpoint of PFS [progression-free survival]. The proportion of participants with undetectable MRD [minimal residual disease] will be summarised (with 95% CIs [confidence intervals]) for participants randomised to consolidation therapy with obinutuzumab at 6 months post-randomisation and then at every time point at which MRD is assessed. CR [complete remission], CRi [complete remission with incomplete marrow recovery] and overall response (at least a PR [partial remission]) will be summarised (with 95% CIs) for participants randomised to consolidation therapy with obinutuzumab at 6 months post-randomisation and then at every time point at which response is assessed. OS [overall survival; the target outcome] and treatment-free survival (TFS) (i.e., time from randomisation to next treatment or death) will both be assessed using Cox proportional hazards models to compare trials arms, adjusting for the minimisation factors. Kaplan-Meier curves, 95% CIs and median survival estimates will also be produced for both analyses by trial arm.”^45^ (We have added the words in square brackets in the example and we recommend their use when reporting the item.)

#### Explanation

This item builds on item 12.3 to inform readers on planned subsequent analyses or studies to verify current findings (on observed benefit, lack of benefit, harm) using a target outcome. Such subsequent analyses or studies include extending follow-up in the current trials to confirm the effect on target outcome(s), surrogate endpoint validation studies, meta-analysis of trials, confirmatory trials, and real world evidence studies, among others. Such subsequent analyses or studies are often not planned or conducted. A survey of cardiovascular trials published in three high impact journals between 1990 and 2011 found that only 27% of trials that used surrogate endpoints as primary outcomes were followed by trials to verify findings in target outcome trials.^87^ In cancer, a retrospective analysis of drug approvals by the US Food and Drug Administration found that despite 56% of accelerated approvals and 37% of traditional approvals not being supported by strong surrogate validation evidence, only 45% of the approvals had subsequent analysis on overall survival—the target outcome.^88^ Such lack of subsequent studies to verify the effect could extend beyond cardiovascular diseases, cancer, and drug related interventions, and could also lead to the continued use of interventions that have no benefit.^23^

Development of the protocol presents an essential stage for trial teams to consider subsequent analyses or studies that can verify the findings of the completed trial. The extension does not mandate authors to conduct subsequent analyses or studies as it depends on feasibility and funding, among other factors. Furthermore, plans to conduct such studies change. Nevertheless, we recommend that authors are transparent in reporting this item (ie, explicit statement of no plans with justification) and describe current plans, including planned follow-up beyond the study period (such as in example 1) or the progress of a postapproval confirmatory trial for protocols describing an accelerated approval trial. This information will allow readers, especially clinicians and intervention end users, to know whether (and when) to expect definitive findings on the effect reported in ongoing or planned trials. Trial authors can update readers on their plans when reporting the trial findings (see CONSORT-Surrogate).

## Section 3d: Methods—monitoring

### Harms (extended)

#### SPIRIT 2013 item 22

Plans for collecting, assessing, reporting, and managing solicited and spontaneously reported adverse events and other unintended effects of trial interventions or trial conduct.

See SPIRIT 2013.^2^

#### SPIRIT-Surrogate extension item 22.1

Comment on whether the trial design (including sample size and follow-up period), given the use of a surrogate endpoint, adequately captures the potential harms of the intervention being tested.

### Example of SPIRIT-Surrogate item 22.1

“As a secondary outcome, we aim to assess the difference in drug-related adverse effects in the two groups; however, the frequency of side effects associated with vancomycin is low, and therefore 200 infants may not be sufficient to detect a difference between the two groups.”^89^

#### Explanation

While trial treatment effects on a surrogate endpoint can indicate a potentially positive impact of an intervention, longer term trial follow-up or introduction of the intervention into routine practice could find the intervention to be harmful on the target outcome.^90^ One widely cited example is the case of suppressing arrhythmia (abnormal heart rhythm), where drugs aimed at reducing arrhythmias as a surrogate endpoint for cardiovascular related mortality were later discovered to increase mortality.^91^ For diabetes, a drug treatment (rosiglitazone) was approved based on its ability to reduce glucose levels as a surrogate endpoint for diabetes complications, cardiovascular risk, and death, but was later linked to higher rates of hospital admission related to heart failure, and of heart attacks.^92^ More recently, a drug (venetoclax) that showed improvement in progression-free survival (a surrogate endpoint for overall survival) was associated with increased mortality in patients with relapsed, refractory multiple myeloma.^93^

The harms could be caused by various factors, including unintended intervention effects not mediated through the surrogate endpoint or known disease causal pathways, or if the intervention does not have a positive impact on the surrogate endpoint despite a positive correlation between the intervention effects on the surrogate endpoint and the target outcome.^90^ ^91^ When a surrogate endpoint is used as the primary outcome, we recommend collecting target outcome data as a part of the primary outcome definition and as secondary outcome(s), because it can provide insights into intervention harms and would override outcomes on the surrogate endpoint. For instance, the BELLINI trial identified higher mortality in the intervention group leading to its early termination: progression-free survival was a primary outcome, and overall survival was a secondary outcome.^93^ ^94^

## Section 4: Ethics and dissemination

See SPIRIT 2013^2^ for item 24 (research ethics approval) and item 25 (protocol amendments).

### Consent or assent (extended)

#### SPIRIT 2013 item 26a

Who will obtain informed consent or assent from potential trial participants or authorised surrogates, and how (see item 32).

See SPIRIT 2013.^2^

#### SPIRIT-Surrogate extension item 26a.1

State whether and how trial participants will be engaged and informed before enrolment that the trial was designed to evaluate an intervention’s effect using a surrogate endpoint.

#### SPIRIT 2013 item 26b (unmodified)

Additional consent provisions for collection and use of participant data and biological specimens in ancillary studies, if applicable.

See SPIRIT 2013.^2^ The following SPIRIT 2013 items are also unmodified: confidentiality (item 27), declaration of interests (item 28), access to data (item 29), ancillary and post-trial care (item 30).

### Example of SPIRIT-Surrogate item 26a.1

“Informed consent: All participants will receive adequate information about the nature, purpose, possible risks, and benefits of the trial [given the use of a surrogate endpoint as the primary outcome] and alternative therapeutic choices using an informed consent protocol approved by the IRB [institutional review board]. All participants must be given ample time and opportunity to ask questions and consider participation in the trial. A completed informed consent form is required for enrolment in the trial. The investigators must maintain the original signed consent form, as well as an additional copy of this form.”^95^ (This example did not implement the item but shows how the item can be reported using the words in square brackets. We recommend that trial teams are explicit on how informed consent was done, such as by research nurses (see SPIRIT 2013^2^).)

#### Explanation

Public engagement (also known as community engagement) involves interacting, listening to, and connecting with members of the public to share research activity or its benefits, discuss relevant issues (such as ethics), or get input on preliminary research ideas.^96^ Patient and public involvement is engagement in a study setting and involves conduct of research with or by members of the public (rather than “for,” “to,” or “about” members of the public).^96^ Public engagement is crucial for trial planning and conduct and also translation of trial findings and increasing benefit for trial participants and the public.^97^ ^98^ Public engagement and informed consent are mutually supportive aspects aimed at the same goal: ensuring conduct of research in a respectful manner and maximising its social value.^99^ ^100^ ^101^

Informed consent is a legal and ethical requirement in all research involving human beings before study participation.^3^ ^102^ It involves adequately informing participants on trial details including the anticipated benefits and potential risks of participation.^3^ ^103^ Therefore, when using surrogate endpoints as primary outcomes, the informed consent process ideally allows for continued engagement of trial participants on the use of surrogate endpoints and their related risks and benefits or start of such an engagement. Nevertheless, current evidence from early phase trials (many of which might rely on surrogate endpoints) suggests that participant risk-benefit communication is suboptimal. In a survey of the informed consent documents from 172 early phase trials, researchers found that only 45% reported the outcome of health benefits (eg, survival, tumour shrinkage), and 63% mentioned the likelihood of health risks, of which 56% were clear on whether risks would be due to research procedures or potentially beneficial interventions.^103^

Inadequate risk-benefit communication calls into question whether consent is fully informed at all. Therefore, informing participants that the trial will use a surrogate endpoint is critical to informed consent.^104^ and public engagement. Notably, participants should be adequately informed on the use of a surrogate endpoint and related limitations (box 3 includes recommendations for the participant information sheet section, with examples). Firstly, participants should be informed in plain language what a surrogate endpoint is and directed towards lay resources on surrogate endpoints, such as blog articles.^105^ ^106^ Secondly, researchers must clarify to participants why a surrogate endpoint is being used, such as to enable faster approval of an intervention and the consequent patient or public benefit. Thirdly, the uncertainty of how well the surrogate endpoint predicts the target outcome should be communicated. Finally, participants should be informed that the trial sample size and follow-up time might not be sufficient to identify all the harms of the intervention under evaluation. This information will allow trial participants to make an informed decision on participation. Additionally, such information will be necessary in some settings for trial participants and the wider public to understand why interventions approved using surrogate endpoints might not inform policy, practice, or reimbursement decisions, because the benefit is not certain. Furthermore, this item builds on the increasing calls for more patient and public engagement and involvement in trials, including in outcome selection^107^ and sharing trial results with the public.^108^

Box 3 **Recommendations for the design of participant information sheet sections informing on the use of surrogate endpoints, with examples**

**Recommendations to structure**

When designing participant information sheets for trials using surrogate endpoints as primary outcomes, trial teams can use the following structure:

- Be explicit that the trial is using a surrogate endpoint, explaining the meaning of a surrogate endpoint to the participants in lay terms; and signpost participants to explanatory resources on surrogate endpoints.
- Inform participants about the practical and positive aspects of using surrogate endpoints, such as getting faster access to treatment.
- Be clear on points of concern on using surrogate endpoints—that is, uncertainty in predicting benefit and limited ability to identify intervention harms.

**Examples of participant information sheet sections implementing the item**

- For a cancer treatment study investigating the benefits of an additional round of immunotherapy (second line) that will help participants’ immune system better fight cancer after previous treatment for cancer treatment:
  “The main study outcome is the disease response (which we will be measured using clinical scanning) and is called a “surrogate endpoint.” This study is not designed to determine if this treatment improves your length and quality of life.
  “By using a surrogate endpoint, this study may enable earlier regulatory approval of the additional immunotherapy and, therefore, faster access for patients, like yourself, to this new treatment. Surrogate endpoints are not always true indicators of how well a treatment works, and studies that use them may also not adequately identify the potential harms of treatment given use of small sample sizes and short follow-up periods.”
- A trial of treatment for heart disease where the main study outcome is the measurement of systolic blood pressure after two months:
  “Measurement of systolic blood pressure is used as a substitute measure for the effects of the study medication on future premature death or blood circulation problems. Such an indirect measure is known as a “surrogate endpoint” and may or may not reflect your health. Current evidence shows that reduction in blood pressure strongly predicts the effect of a drug on reduction in your future risk of heart disease complications.
  “Using indirect measures may provide evidence that allows sooner regulatory approval and, therefore, faster access for patients, like yourself, to this new treatment. However, such measures are not always true indicators of how well treatment works and may also not capture the potential harms of treatment cure given that they are used in smaller and quicker studies.”

The way this item will be implemented will not be a one-size-fits-all approach. Trial investigators and recruitment staff must carefully consider how best to engage participants on surrogate endpoints and their use in the trial. These conversations might be difficult, given the historical reliance on and the assumption that surrogate endpoints predict benefits in some disease areas.^109^ They might also be confusing for participants when intermediate outcomes are used that could have some perceived benefit but are still surrogate endpoints for target outcomes.^29^ Conversely, the engagements could be insightful, with participants sharing what they consider important. For example, community engagement in a recent trial aimed at evaluating treatments for achondroplasia found that while some community members felt that height increase (a surrogate endpoint) was of less priority than better health (the target outcome), some participants considered that increased height was also important because it has benefits such as improved self-esteem and reduced discrimination.^110^ In summary, the conversations (as part of the public engagement) should provide insightful perspectives on what is important to participants but could also mean more time is spent during consenting; and a possible risk of reduced willingness to participate in the trial. Nevertheless, these conversations are possible, necessary, and timely, and guarantee actual informed consent and public engagement.

For trials that do not implement this item, authors should be explicit about it and provide a justification. Furthermore, implementing this item is generally novel, and even in trials not specific to surrogate endpoints, more research is needed to understand aspects of risk-benefit communication, including the balance between overpromising and overpessimism on anticipated benefits, rationale and extent of health benefits, the likelihood of approval of drugs under study, and balance between too little and too much information on risk-benefit.^103^

## Dissemination policy

### SPIRIT 2013 item 31c (extended)

Plans, if any, for granting public access to the full protocol, participant level dataset, and statistical code.

See SPIRIT 2013.^2^

### SPIRIT-Surrogate extension item 31c.1

If surrogate and target outcome data will be collected in the trial, state the open access arrangements for the data for future secondary research.

### Examples of SPIRIT-Surrogate item 31c.1

“We will share individual patient data within 2 years after the trial is completed, and the original data will be collected using a clinical recording formula (both paper and electronic versions).”^111^

#### Explanation

As emphasised previously, the collection of target outcome data is vital when a surrogate endpoint is used as the primary outcome. Both datasets can be used for surrogate endpoint validation, and target outcomes could be used to monitor intervention harms. Therefore, we encourage research teams to consider collecting target outcomes as secondary measures. Although surrogate endpoint validation is essential, it can be resource and time intensive, because it requires the availability of both surrogate endpoint and target outcome data.^20^ The challenge is compounded by limited access of individual participant data from completed studies for secondary research.^112^ Therefore, when both datasets are collected, data sharing enables leveraging it for secondary research including surrogate endpoint validation.

Statements that data will be made available are not sufficient in implementing this item. Trial teams should have a genuine commitment to sharing their datasets. Recent surveys of published trials revealed that access to individual patient level data was very limited, with less than 25% of trial teams providing access, despite most trial authors declaring their intention to share the data.^113^ ^114^ Several challenges to data sharing exist, including concerns about participant confidentiality, perceived risks of inappropriate data use, and competition from peers who have access to the data.^113^ ^115^ Therefore, in cases where data sharing (for part or all the data) is not feasible, authors should explicitly state the reasons and provide a justification for their decision.

## Conclusion

The SPIRIT-Surrogate extension sets out the minimum requirements for reporting trial protocols where surrogate endpoints are used as the primary outcomes. We recommend using the SPIRIT-Surrogate extension alongside the main SPIRIT 2013 reporting guideline. By following this extension, researchers will improve trial transparency, reduce research waste, and ultimately this will benefit healthcare and population health.

We recommend that all stakeholders, including funders, ethical reviewers, regulators, journal editors and peer reviewers, promote using the SPIRIT-Surrogate extension whenever applicable. Nevertheless, using extensions does not eliminate other sources of research waste, such as selecting the wrong research question, biases, or poor study design.^4^ Notably, trial teams and readers should be aware that biases in measuring surrogate endpoints contribute to inaccurate predictions of the intervention effects.^20^ Finally, adequate reporting of all items specified in this extension does not preclude trial teams and the wider scientific community from evaluating the same interventions based on the target outcomes, whenever possible.

## Data Availability

Additional data are available through request from the corresponding author. After publication of all project’s manuscripts, data will be deposited in the UK Data archive, and will be accessed through their standard end user licence (this would require users to login to the UK Data Service).

## Web extra

Extra material supplied by authors

Web appendix 1: Supplementary material

Web appendix 2: Combined SPIRIT-Surrogate extension checklist

Web appendix 3: Acknowledgments

## Notes

Contributors: AMM, RST, and OC are joint first authors. PD, CJW, AEY, RST, and OC were involved in funding acquisition. AMM, PD, DS, CJW, AEY, RST, and OC were involved in study conception and design. AMM, PD, DS, CJW, AEY, JB, RST, OC, NJB, SB, A-WC, GSC, DD, MOf, MOu, and JSR contributed to the methodology. NJB, SB, A-WC, GSC, DD, MOf, MOu, and JSR supervised the project. AMM, RST, and OC curated the data and conducted formal analysis. AMM, RST, OC, SM, DS, RH, and AW were responsible for the first draft of the manuscript. All authors critically reviewed the first draft and approved the final version. AMM, RST, and OC are the guarantors. The corresponding author confirms that all listed authors meet authorship criteria and that no others meeting the criteria have been omitted.

Transparency: The lead authors (the manuscript’s guarantors) affirms that this manuscript is an honest, accurate, and transparent account of the study being reported; that no important aspects of the study have been omitted; and that any discrepancies from the study as planned (and, if relevant, registered) have been explained.

Patient and public involvement: Four patient and public involvement (PPI) partners (DS, SM, RH, AW) were consensus meeting delegates and are coauthors of this extension. DS has been a member of the project team and the project PPI lead. Nineteen PPI partners participated in the e-Delphi survey.

Dissemination to participants and related patient and public communities: The SPIRIT-Surrogate and CONSORT-Surrogate extensions will be disseminated to the public through press releases, presentations at conferences, video tutorials, and plain language summaries posted on websites and social media.

Provenance and peer review: Not commissioned; externally peer reviewed.
