# Supplementary material for: Reporting of surrogate endpoints in randomised controlled trial protocols (SPIRIT-Surrogate): extension checklist with explanation and elaboration
Source: BMJ. 2024 Jul 9;386:e078525. doi: 10.1136/bmj-2023-078525 (PMC11231880; doi:10.1136/bmj-2023-078525)
Supplement: Supplementary file 3 — Web appendix 3: Acknowledgments [file mana078525.ww3.pdf]

Acknowledgements

Professional organisation and networks

We acknowledge all professional organisations and networks who helped in mobilising participants for the e-Delphi survey through posting calls in their websites, social media accounts, newsletters or sending invites to their mailing lists. Below are the organisations and networks who confirmed helping with our mobilisation efforts.

Cancer Research UK Clinical Trial Units; UK Renal Trials Network; Cochrane Bias Methods Group; European Association of Science Editors (EASE); European Clinical Research Infrastructure Network (ECRIN); Health Research Board Trials Methodology Research Network (HRB-TMRN); International Behavioural Trials Network (IBTN); International Clinical Trial Centre Network; International Network of Agencies for Health Technology Assessment (INAHTA); International Society for Medical Publication Professionals (ISMPP); MRC-NIHR Trial Methodology Research Partnership (TMRP); Network of Networks (N2); Pan-Asian Resuscitation Outcomes Study (PAROS); Society of Clinical Trials (SCT); Swiss Clinical Trials Empirical Assessment & Methods (STEAM); UK Trial Managers' Network; UKCRC Registered CTU Network; World Association of Medical Editors (WAME).

Participants in e-Delphi survey

We acknowledge all the participants who took part in either rounds of the e-Delphi survey. Their participation does not imply they agree with overall ratings of items or items included in the extension. We acknowledge all in this list in alphabetical order of their first names.

Aad van der Lugt; Aaron Lawson McLean: Abhik Das; Abraham Samuel Babu; Achilles Thoma; Alain Amstutz; Alastair O'Brien; Alexandra Barr; Alparslan Turan; Amanda Adler; Amanda Roberts; Ana Marusic; Andreas Lundh; Andrew Jull; Andrew Worrall; Angela Casbard; Angèle Gayet-Ageron; Ann Russell; Arsenio Paez; Atara Ntekim; Baljit Singh; Beatriz Flores; Benjamin Davies; Brennan Kahan; Brigid Gillespie; Caroline McCarthy; Caroline Schmutz; Catey Bunce; Chara Kani; Cheow Peng Ooi; Chris Weir; Christelle Nguyen; Christian Gluud; Christina Danielli Coelho de Morais Faria; Christina Yap; Christopher Leptak; Chris Sandford; Cinara Sacomori; Clifton Fuller; Colin Berry; Cory Goldstein; Deeksha Gibran; Desirée van der Heijde; Daniel Gladwell; Daniel Reim; Daniela Raggio; David McAllister; David Tom Huang; David Wheeler; Declan Devane; Derek C Stewart; David Lora Pablos; Diana R Elbourne; Edoardo la Sala; Elena Biagioli; Eliana Rulli; Elizabeth Garrett-Mayer; Emily Lam; Emmanouil Zouridakis; Eric Frison; Esme Radin; Eva Broeckelmann; Falk Schwendicke; Francesco Perrone; Gary Collins; Gavin Lawler; Gayathri Delanerolle; Geert Molenberghs; Giulio Formoso; Gregory Curfman; Greta Castellini; György Németh; Haitao Pan; Hector Rojas-Anaya; Helen Mohan; Helene Wellington; Huseyin Naci; Ina Jochmans; Irene De Simone; Jacqui Gath; Jake Emmerson; Jan Adolfsson; Jane Blazeby; Jeanne Lenzer; Jean-Pierre Boissel; Jeffrey Probstfield; Jeppe Lerche la Cour; Jianrong Zhang; Jo Haviland; Joel Lexchin; John H Powers; John Petrie; Jose Quebral; Joshua Savage; Katja Suter; Kent Johnson; Kieran O'Brien; Kim Bennell; Kim Madden; Kim Papp; Kimberly Ong; Kirsty Rhodes; Kukuh Noertjojo; Larisa Tereshchenko; Lars Hemkens; Laura Richert; Laurence Freedman; Leen Verleye; Lindsay Jibb; Lisa Askie; Lisa Campbell; Lisa Stamp; Lise Hestbaek; Lorenzo Emigrato; Luca Bertolaccini; Luca Giovanni Campana; Lucia Arellano; Lucio Marinelli; Ludovic Trinquart; Madeleine Clout; Malcolm West; Manohara Halasiddappa; Marc Froissart; Maria José Martinez-Zapata; Maria Carmela Leo; Marianna Mitratza; Mario Ouwens; Marissa Lassere; Mark Siedner; Martin Underwood; Martina Messow; Mary Dunne; Masha Kocherginsky; Matt Stevenson; Maureen Smith; Michela Cinquini; Michelle Ghert; Mike Clarke; Molly E. Hoke; Nicholas Latimer; Niels Stens; Nurulamin Noor; Oriana Ciani; Orlando Guntinas-Lichius; Pau Alcubilla; Paul

Tappenden; Paula Brauer; Pawin Numthavaj; Péron Julien; Peter Tugwell; Peter Craig; Peter Gotzsche; Philip Pallmann; Qian Shi; Quentin A Hill; Ray Harris; Rebecca Grainger; Rebecca Mister; Rejina Verghis; Rob Jones; Robert Golub; Robin Christensen; Rod Taylor; Roger Wilson; Rohini Sharma; Rosario R. Ricalde; Rossella Di Bidino; Roy Beck; Rui Duarte; Salvatore De Masi; Sara Tedeschi; Sarah Markham; Sarah Moore; Shalini Murthy; Sharleen O'Reilly; Shawna Grosskleg; Silvia Gianola; Simon L Bacon; Simon McNamara; Steven Julious; Steven M Snapinn; Subhash Pokhrel; Susan S Ellenberg; Tassos C Kyriakides; Tero Kortekangas; Timothy Feeney; Tiziano Innocenti; Vincent van Vugt; Vincenzo Bagnardi; Vinicius Pedrazzi; Vito Domenico Bruno; Wang Pok Lo; Will Wilson; Yagiz Uresin; Yousef Rezaei; Zoe Craig; Zoe Moodie

Participants in the piloting of the extension

We acknowledge all the participants who took part in the piloting of the SPIRIT-Surrogate. We acknowledge all in this list in alphabetical order of their first names.

Eric Frison; Felix Gerber; Kim Madden; Marita Olsson; Sharleen O'Reilly; Simon L Bacon
